# UNDERSTANDING CHALLENGES & ADAPTIVE STRATEGIES FOR BATHING & SHOWERING AMONG ADULTS WITH MOBILITY DISABILITIES

**DOI:** 10.1093/geroni/igae098.4366

**Published:** 2024-12-31

**Authors:** Pallabi Bhowmick, Elena Remillard, Meghana Daliparthi, Kainat Shahid, Wendy Rogers, Ian Rice

**Affiliations:** University of Illinois Urbana-Champaign, Champaign, Illinois, United States; Georgia Institute of Technology, Atlanta, Georgia, United States; University of Illinois Urbana-Champaign, Champaign, Illinois, United States; University of Illinois Urbana-Champaign, Champaign, Illinois, United States; University of Illinois Urbana-Champaign, Champaign, Illinois, United States; University of Illinois Urbana-Champaign, Champaign, Illinois, United States

## Abstract

Self-care and bathing activities are among the most challenging and dangerous activities for individuals aging with mobility disabilities, yet their technology needs remain largely unexplored. There is a need for more specificity about the nature of the challenges. Two user needs studies were conducted exploring challenges and strategies for everyday activities among individuals with long-term mobility impairments (lasting at least 10 years). We analyzed data on bathing/showering challenges from a subsample of participants from these two studies (n=28), aged 52–86 (M=64; SD=9.2), who were wheelchair users and were unable to stand due to lower body mobility impairments. Findings reveal several critical issues and adaptive strategies around bathing/showering. 82% of the participants either stopped bathing (i.e., submerging in a tub) or could not bathe without assistance, relying instead on overhead shower systems and adaptations like roll-in, no-threshold showers, grab bars, and shower chairs/benches. Secondly, the majority transitioned to handheld showerheads over fixed showerheads, and to using bathing wands/reacher sticks for greater flexibility in cleaning themselves. Lastly, several participants expressed concerns about the loss of upper body strength with age, making it difficult to use heavy showerheads or adjust water temperature with controls that are difficult to reach and press. These findings underscore the need for customizable, assistive showering devices for individuals who must shower in a seated position, a research area that has received limited attention. Designing a lightweight robotic showerhead with accessible controls could be a promising solution, which would significantly enhance independence and quality of life for these individuals.

